# The Effect and Persistency of 1% Aluminum Chloride Hexahydrate Iontophoresis in the Treatment of Primary Palmar Hyperhidrosis

**Published:** 2011

**Authors:** Khosro Khademi Kalantari, Afsane Zeinalzade, Farzad Kobarfard, Salman Nazary moghadam

**Affiliations:** a *Department of Physiotherapy, Faculty of Rehabilitation, Shahid Beheshti University of Medical Sciences, Tehran, Iran.*; b *Department of Medicinal Chemistry, School of Pharmacy, Shahid Beheshti University of Medical Sciences, Tehran, Iran.*; c *Faculty of Rehabilitation, University of Social Welfare and Rehabilitation Sciences (USWRS), Tehran, Iran.*

**Keywords:** Iontophoresis, Aluminum chloride, Palmar hyperhidrosis, Hexahydrate

## Abstract

Topical solutions containing aluminum chloride are known to be the first line of therapy for hyperhidrosis. Palmar hyperhidrosis however, is less responsive to aluminum chloride therapy and successful treatment may require 6-8 h application of high concentrations up to 30% that commonly leads to skin irritation. The purpose of this study is to investigate the effect of 30 min iontophoretic application of low concentration (1%) aluminum chloride solution in patients with palmar hyperhidrosis.

Iontophoresis of 1% aluminum chloride was applied to one hand of twelve patients with palmar hyperhidrosis for four successive days. The subjects’ other hand was treated topically with the same solution at the same time. Gravimetric and iodine- starch tests were performed at baseline, 3 days, 1, 2, 3 and 4 weeks after the last treatment. Experimental hand showed significant hypohidrosis from the 3^rd^ day until the 4^th^ week post-treatment (p < 0.04) which was lower than the control hand throughout the follow-up period. Iontophoresis of low concentration aluminum chloride hexahydrate can induce hypohidrosis that is more persistent than its topical application and with no side effects.

## Introduction

Primary focal hyperhidrosis is an idiopathic disease defined by excessive sweating exceeding the needs of thermoregulation that typically affects the axillae, palms, soles, and face. In most of the cases, there is no underlying disease and generally it is worsened by emotional stress rather than heat or exercise (1). The primary defect in patients with hyperhidrosis may be hypothalamic hypersensitivity to emotional stimuli from the cerebral cortex (2). The disorder, which affects up to 1% of the general population, is associated with considerable physical, psychosocial, and occupational impairments. Current therapeutic strategies include topical aluminum salts, tap-water iontophoresis, application of anticholinergic agents such as Botulinum toxin, local surgical approaches, and sympathectomies. These treatments, however, have been limited by a relatively high incidence of adverse effects and complications.

Aluminum chloride is commonly used topically as the first-line therapy for primary hyperhidrosis (PHH) (3). Several studies have shown that aluminum salts can cause an obstruction of the distal sweat gland ducts (4, 5). A mechanism underlying this obstruction has been proposed as: the metal ions precipitate with mucopolysaccharides, damaging epithelial cells along the lumen of the duct and forming a plug that blocks sweat output. While it is used in regular antiperspirants, hyperhidrosis sufferers need a much higher concentration to be able to treat the symptoms of the condition effectively. A 15% aluminum chloride solution or higher usually takes about a week of nightly use to stop the axillary sweating, with one or two nightly applications per week to maintain the results (6). The solution is usually not effective for palmar (hand) and plantar (foot) hyperhidrosis (7) and successful treatment may require 6-8 h application of high concentrations up to 30% (8). An aluminum chloride solution can be very effective; some people, however, cannot tolerate the dermatitis that the high concentration solution can cause. 

Although the ionic composition of aluminum chloride solution and the suggested mechanism of its effect make it suitable for iontophoresis, the efficiency of this method has not been investigated yet. Therefore, we investigated the efficacy and persistency of hypohidrosis induced by low concentration of aluminum chloride hexahydrate (1%) delivered by 30 min iontophoresis in patients with primary palmar hyperhidrosis.

## Experimental

Twelve subjects (6 females and 6 males) with primary hyperhidrosis, aged 20-32 years (23.17 ± 3.6) participated in this experimental study. Patients were selected and referred according to the following criteria by a dermatologist: 

Excessive palmar perspiration and excessive sweating for more than one year. 

Patients with organic disease, such as hyperthyroidism were excluded in this study. Informed consent was obtained from all patients after full written and oral explanation. The research followed the tenets of the Declaration of Helsinki promulgated in 1964 and was approved by the ethics committee of Shahid Beheshti University of Medical Sciences.

Iontophoresis of aluminum chloride hexahydrate was applied to one hand, selected randomly from the dominant and non-dominant side (5 non-dominant and 7 dominant hands), using a galvanic stimulator (Enraf Nonius, Dynatron 438, Netherlands) with the intensity of 12 mA for 30 min. The iontophoretic treatment was repeated for four successive days. The other hand was regarded as control and treated with topical method. The active electrode covered the palmar surface of the hand from metacarpophalangeal crease to distal wrist crease. The indifferent electrode was placed on the anterior surface of the forearm. Anode was selected as the active electrode. In this study, 1% aluminum chloride hexahydrate solution in ethyl alcohol was used for treatment. This low concentration of aluminum salt was selected to curtail the possible side effects (9). Twenty milliliter of solution was delivered by iontophoresis to the experimental hand in each session. A thin layer of absorbent pad was used to retain the solution under the electrodes. The same amount of solutions was also used for the control hand and the remaining was washed out after 30 min.

All patients underwent one pre-treatment and 5 post-treatment evaluation at 3 days, 1, 2, 3 and 4 weeks after the final treatment session. In each evaluation session, they were acclimatized in a temperature and humidity controlled room (temperature 25°C and relative humidity 60%) for 15 min. Gravimetric and iodine-starch tests were performed to evaluate the sweat production rate. For the gravimetric test, we used a standardized filter paper that was weighed on a high-precision laboratory scale (with the accuracy of 0.0001 g). The patient’s hand was then placed on the paper while the forearm was rested on the table with flexion of the elbow in order to remove the weight of the upper limb. After exactly 1 min, the paper was weighed again, indicating the rate of sweat secretion milligrams per min. The iodine-starch test consisted of painting a solution made up of 2 g of iodine and 4 g of potassium iodine in alcohol to 100 mL over the skin of the hand and fingers. After being dried, a fine starch powder was applied and after 2, 5 and 8 min digital photos were taken. Sweat causes the color of the powder to turn dark blue. A repeated-measures ANOVA with post-hoc Bonferroni test was used to compare the sweat intensities at different evaluation sessions. 

## Results and Discussion

**Figure 1:**
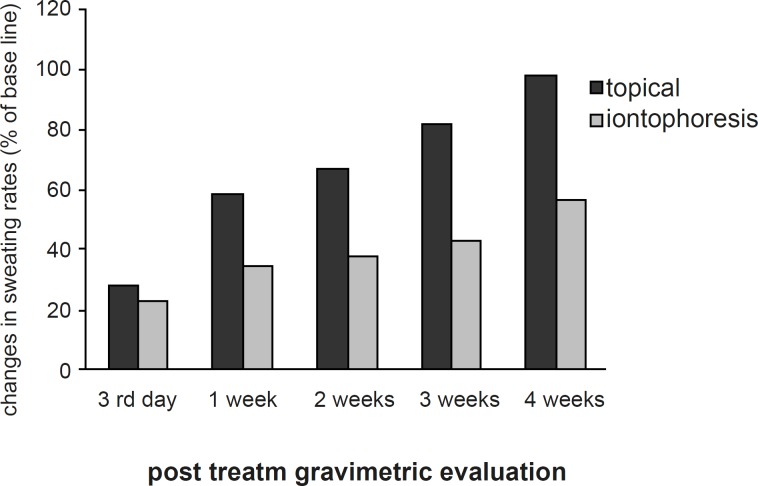
Changes of sweating rates at all post-treatment evaluation sessions

The sweating rate of the control and experimental hands showed no significant difference at the base line. The mean sweating rate was reduced in both hands post-treatment. In experimental hand, the reduction was statistically significant at 3^rd^ day, 1^st^, 2^nd^, 3^rd^ and 4^th^ week (p < 0.04) after the treatment (Figure 1). 

**Figure 2:**
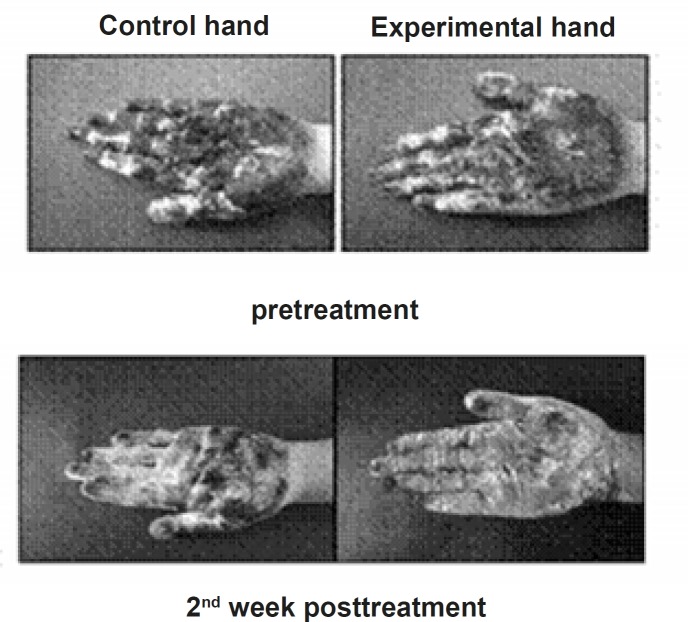
Iodine starch test of the experimental and control hand before and 2 weeks after the treatment.

In the control hand, however, the sweat reduction was only significant statistically until the 1^st^ week post-treatment (p < 0.05). The least sweating rate was at 3rd day and 1^st^ week after the treatment in the experimental hand (mean reduction of 77% and 65% respectively) and 3^rd ^day after the treatment in the control hand (mean reduction of 72%). The mean sweating rate of the experimental hand was lower than the control hand at all post-treatment evaluations, however, the differences between the hypohidrosis induced by iontophoresis and topical application of aluminum chloride became statistically significant after the 1^st^ week (p < 0.03) (Figure 2).

No skin irritation was observed during the trial except for one subject who experienced a mild and transient itching sensation.

An iontophoretic application of as low as 1% aluminum chloride hexahydrate is shown to considerably reduce the palmar sweating rate with long endurance and least skin irritation compared to topical method. The possibility of using low concentration of aluminum chloride solution and the short-time exposure to it are the merits for iontophoretic application of the solution. 

presently, various treatments have been applied to treat focal primary hyperhidrosis, such as antiperspirants (10), electrophoretic methods (11), administration of botulinum toxin to the vicinity of the lesion (11, 12) and sympathectomy (13). Topic antiperspirants containing aluminum chloride, are known to be the first line of therapy for axillary hyperhidrosis, however, they are ineffective in the treatment of palmar or plantar hyperhidrosis as the skin is much thicker (7, 8). A successful treatment for palmar hyperhidrosis may require 6-8 h application of high concentrations up to 30% (8). Skin irritation is the main side effect of treatment with aluminum salt and despite all precautions, can still be high (4). The high incidence of dermatitis is attributed to the high concentration of the solution and the long duration of exposure that is required for the desired effect.

Iontophoresis is a recognized therapeutic method for delivering ionic and also neutral compounds, *i.e. *drugs, into and through the skin by applying electrical current. Iontophoresis enhances the transdermal delivery of ionized drugs through the skin›s outermost layer (stratum corneum) which is the main barrier to drug transport. The absorption rate of the drug is increased, however, once the drug passes through the skin barrier, natural diffusion and circulation are required to shuttle the drug to its proper location. The mechanism, by which the iontophoresis works, is based upon the knowledge that similar electrical charges repel. Applying a positive current from an electrode to a solution applied to a skin surface, will drive the positively charged drug ions away from the electrode and into the skin. Obviously, negatively charged ions will behave in the same manner. In iontophoresis, the transmission of drugs and chemicals through the body is forced by direct electrical current with specific magnitude and duration (11). Although iontophoresis has been used for many years to deliver drugs through the skin and the aluminum chloride is considered as an effective treatment for palmar hyperhidrosis with a wide range of use in dermatological practice (8, 14), no study has been published yet about the iontophoretic application of pure aluminum salt in treatment of focal hyperhidrosis. In the only published study, a combination of glycopyrrolate and aluminum chloride was delivered by iontophoresis for the treatment of patients with palmar hyperhidrosis (9) which raises the ambiguity of the contribution of each element to the outcome of this experiment.

Different mechanisms of iontophoretic transport for ionic and neutral permanents have been suggested as electrophoresis and electroosmosis, respectively. When a low electric field is applied across the human epidermal membrane, the transport enhancement of ionic permeant in the preexisting pores of the stratum corneum is the result of changes in the observed behavior of membranes following the electrophoresis. In addition to the electrophoresis in the preexisting pores, there can generally be a significant (10 to 100 fold enhancement) contribution to transport enhancement arising from new pore induction (electroporation) (15), changes in pore sizes and flux in shunt pathways (16) (*e.g. *hair follicles and sweat glands). Sweat glands (large pores) in skin traverse the stratum corneum, epidermis and dermis and are known to act as the most efficient shunt pathways for the entry of any drugs delivered through the skin (17). The fact that the sweat glands are the main route of entry for drugs implies that iontophoresis can effectively enforce the aluminum salt to penetrate directly into the sweat glands. The common mechanism suggested for the effect of topical aluminum salt (4) and direct electrical current administration (18) is the occlusion of sweat glands and this would justify the application of the aluminum salt by iontophoresis. 

The method used in this study consisted of two effective elements; direct current and aluminum salt. Each of them is considered effective in the treatment of hyperhidrosis. Applied direct current alone can reduce the sweating rate, however, continuous application is required since the results are often short-lived (few days) and they may be insufficient (19, 20). Topical aluminum chloride applied to the control hand in present experiment, also induced significant hypohidrosis but still with low endurance. It can be concluded that the induced long-lasted hypohidrosis in the present experiment is most likely related to the accumulative effect of both elements contributed in iontophoresis of aluminum salt. This could result in more intense and long-lasting occlusion of the sweat glands in the iontophoretic hand compared to the control one.

Iontophoretic application of aluminum salt can be considered as a non-invasive and a safe alternative treatment method especially in the patient with palmar hyperhidrosis and sensitive skin who can not tolerate the long-period contact with topical solutions.
